# Lung transplantation following controlled hypothermic storage with a portable lung preservation device: first multicenter European experience

**DOI:** 10.3389/fcvm.2024.1370543

**Published:** 2024-06-06

**Authors:** An-Lies Provoost, Rene Novysedlak, Dirk Van Raemdonck, Jan Van Slambrouck, Elena Prisciandaro, Christelle M. Vandervelde, Annalisa Barbarossa, Xin Jin, Karen Denaux, Paul De Leyn, Hans Van Veer, Lieven Depypere, Yanina Jansen, Jacques Pirenne, Arne Neyrinck, Sofian Bouneb, Catherine Ingels, Bart Jacobs, Laurent Godinas, Laurens De Sadeleer, Robin Vos, Monika Svorcova, Jaromir Vajter, Jan Kolarik, Janis Tavandzis, Jan Havlin, Zuzana Ozaniak Strizova, Jiri Pozniak, Jan Simonek, Jiri Vachtenheim, Robert Lischke, Laurens J. Ceulemans

**Affiliations:** ^1^ Department of Thoracic Surgery, University Hospitals Leuven, Leuven, Belgium; ^2^ Laboratory of Respiratory Diseases and Thoracic Surgery (BREATHE), Department of Chronic Diseases and Metabolism, KU Leuven, Leuven, Belgium; ^3^ Prague Lung Transplant Program, 3rd Department of Surgery, First Faculty of Medicine, Charles University and Motol University Hospital, Prague, Czech Republic; ^4^ Laboratory of Abdominal Transplantation, Transplantation Research Group, Department of Microbiology, Immunology and Transplantation, KU Leuven, Leuven, Belgium; ^5^ Department of Abdominal Transplant Surgery and Transplant Coordination, University Hospitals Leuven, Leuven, Belgium; ^6^ Department of Anesthesiology, University Hospitals Leuven, Leuven, Belgium; ^7^ Department of Cardiovascular Sciences, Anesthesiology and Algology, KU Leuven, Leuven, Belgium; ^8^ Clinical Division and Laboratory of Intensive Care Medicine, Department of Cellular and Molecular Medicine, KU Leuven, Leuven, Belgium; ^9^ Department of Respiratory Medicine, University Hospitals Leuven, Leuven, Belgium; ^10^ Department of Anesthesiology and Intensive Care Medicine, Second Faculty of Medicine, Charles University and Motol University Hospital, Prague, Czech Republic; ^11^ Department of Immunology, Second Faculty of Medicine, Charles University and Motol University Hospital, Prague, Czech Republic

**Keywords:** controlled hypothermic storage, lung preservation, overnight bridging, preservation temperature, preservation time, primary graft dysfunction, total ischemic time

## Abstract

**Introduction:**

Compared with traditional static ice storage, controlled hypothermic storage (CHS) at 4–10°C may attenuate cold-induced lung injury between procurement and implantation. In this study, we describe the first European lung transplant (LTx) experience with a portable CHS device.

**Methods:**

A prospective observational study was conducted of all consecutively performed LTx following CHS (11 November 2022 and 31 January 2024) at two European high-volume centers. The LUNGguard device was used for CHS. The preservation details, total ischemic time, and early postoperative outcomes are described. The data are presented as median (range: minimum–maximum) values.

**Results:**

A total of 36 patients underwent LTx (i.e., 33 bilateral, 2 single LTx, and 1 lobar). The median age was 61 (15–68) years; 58% of the patients were male; 28% of the transplantations had high-urgency status; and 22% were indicated as donation after circulatory death. In 47% of the patients, extracorporeal membrane oxygenation (ECMO) was used for perioperative support. The indications for using the CHS device were overnight bridging (*n* = 26), remote procurement (*n* = 4), rescue allocation (*n* = 2), logistics (*n* = 2), feasibility (*n* = 1), and extended-criteria donor (*n* = 1). The CHS temperature was 6.5°C (3.7°C–9.3°C). The preservation times were 11 h 18 (2 h 42–17 h 9) and 13 h 40 (4 h 5–19 h 36) for the first and second implanted lungs, respectively, whereas the total ischemic times were 13 h 38 (4 h 51–19 h 44) and 15 h 41 (5 h 54–22 h 48), respectively. The primary graft dysfunction grade 3 (PGD3) incidence rates were 33.3% within 72 h and 2.8% at 72 h. Intensive care unit stay was 8 (4–62) days, and the hospital stay was 28 (13–87) days. At the last follow-up [139 (7–446) days], three patients were still hospitalized. One patient died on postoperative day 7 due to ECMO failure. In-hospital Clavien–Dindo complications of 3b were observed in six (17%) patients, and 4a in seven (19%).

**Conclusion:**

CHS seems safe and feasible despite the high-risk recipient and donor profiles, as well as extended preservation times. PGD3 at 72 h was observed in 2.8% of the patients. This technology could postpone LTx to daytime working hours. Larger cohorts and longer-term outcomes are required to confirm these observations.

## 1 Introduction

Optimal donor lung preservation is a critical determinant of successful lung transplantation (LTx). For decades, static ice storage (SIS) has been the standard for organ preservation. However, the vulnerability of lung tissue to cold-induced injury caused by near-freezing temperatures may increase the risk of severe primary graft dysfunction (PGD) (1–3). Moreover, preservation on ice is limited to a maximum of 8 h. Therefore, SIS constrains the donor pool and frequently necessitates LTx to be performed overnight under suboptimal working conditions with limited staff. The flaws of SIS have fueled a renewed interest in optimizing donor lung preservation to further improve outcomes after LTx (3–10). Pioneering animal research in the 1990s, followed by the first recent clinical application in Toronto, showed that controlled hypothermic storage (CHS) at 10°C better preserves mitochondrial integrity (3, 4, 6, 8, 11–14). This approach effectively maintains tissue metabolism and mitigates lung injury during the interval between procurement and implantation. Furthermore, CHS enables the prolongation of preservation times, facilitating a shift to planned semi-elective transplant procedures and expanding the donor pool by increasing geographic reach and optimizing donor–recipient matching.

The portable device for CHS (LUNGguard™) maintains the temperature between 4 and 8°C (15). The system was used for the first time in North America in February 2021 (at Duke University Hospital, North Carolina, USA), and introduced in Europe on 11 November 2022 (with the first European LTx performed using the device at the University Hospitals Leuven, Belgium) (16, 17). No manuscripts on the clinical outcome have been published, to our knowledge. The aim of this study is to provide a descriptive cohort analysis of the first European experience on lung CHS with this device by reporting the perioperative characteristics and short-term outcome.

## 2 Methods

### 2.1 Study design

A prospective observational study of all consecutive cases that underwent LTx between 11 November 2022 and 31 January 2024 was conducted at two European high-volume centers: (1) the University Hospitals Leuven (Leuven, Belgium) and (2) the Motol University Hospital (Prague, Czech Republic). The sole inclusion criterion was lung preservation with the CHS device, and there were no exclusion criteria. Data were collected from written and electronic patient files, as well as donor data prospectively collected by Eurotransplant. The study was approved by the research Ethics Committees of Leuven (S67697) and Prague (EK387/23). Written informed consent was obtained from each patient. The follow-ups were reported until 31 January 2024.

### 2.2 Controlled hypothermic storage

All lungs were stored in a portable CHS device (LUNGguard) developed by Paragonix Technologies (Waltham, MA, USA). The SherpaCool phase-changing technology enables CHS by maintaining preservation temperatures at 4–8°C for 40 h. The system received the Food and Drug Administration (FDA) clearance in the United States, and the CE (for Conformité Européenne or European Conformity) mark in Europe. A smartphone application connected to a logger and thermometer in the CHS device permits remote continuous real-time monitoring of location and preservation temperature (15).

Routinely, during procurement the lungs were flushed in an antegrade fashion: in Leuven, 4 L OCS™ lung solution (TransMedics, Inc., Andover, MA, USA) was used, whereas in Prague, 6 L Perfadex™ (XVIVO AB, Göteborg, Sweden). After procurement the lungs were split at the donor center and additionally flushed in a retrograde fashion with 0.5–1 L per lung. A maximum of 250 g of ice was used on the bench table, and in Leuven the donor lung surface temperature was controlled by infrared thermal camera prior to storage. Next, the lungs were packed separately in three plastic bags as in our standard approach, and stored in the CHS device: the first bag included the organ itself immersed in 1 L of preservation solution, the second bag was filled with 1 L saline and the first bag, and the third bag contained the first two bags without additional solution. The preservation solution and saline were stored in a fridge at 6°C at the recipient center, and were placed in the CHS device for transport to the donor center, and only removed from the CHS device just prior to its utilization. Finally, after packing, the lungs were stored simultaneously in the CHS device, and the storage temperature was measured continuously through the built-in thermometer of the device.

The clinical protocol for overnight bridging gradually changed with growing experience. At first, lung preservation was only extended with the CHS device for cases with expected cold-flush after 10:00 PM and with recipient anesthesia at 7:30 AM. Eventually, the window of extended preservation was prolonged to cases with expected cold-flush after 6:00 PM.

### 2.3 Recipient, donor, and procedural variables

The recipient characteristics included the recipient center (Leuven, Prague), sex (male, female), age, body mass index (BMI), indication for LTx [chronic lung allograft dysfunction bronchiolitis obliterans syndrome (CLAD-BOS), chronic obstructive pulmonary disease (COPD), cystic fibrosis (CF), interstitial lung disease (ILD), pulmonary vascular disease (PVD)], time on waiting list, high-urgency (HU) listing, duration of preoperative hospitalization, and need for preoperative extracorporeal membrane oxygenation (ECMO).

Donor characteristics were sex (male, female), age, BMI, type of donation [donation after brain death (DBD), donation after circulatory death (DCD)], cause of brain injury (cardiac arrest, cerebral ischemia, intracerebral hemorrhage, status epilepticus, suicide, trauma), intensive care unit (ICU) stay, partial arterial oxygen pressure over the fraction of inspired oxygen [partial oxygen pressure (PaO2)/fraction of inspired oxygen (FiO2)], Oto-score for secretions (0 = none, 1 = minor, 2 = moderate, 3 = major), and Oto-score for chest x-ray findings (0 = clear, 1 = minor, 2 = opacity 1 ≤ lobe, 3 = opacity > 1 lobe) (18).

Lung preservation variables included indication for CHS, preservation temperature, preservation time for the first and second implanted lung, distance between donor and recipient center, and mode of transport. Preservation time was defined as the interval between the moment the lungs were inserted and removed from the CHS device.

The surgical variables were type of LTx (single, bilateral, lobar), surgical approach (anterolateral thoracotomy, clamshell), need and indication for intraoperative ECMO, blood product transfusion (packed cells, fresh frozen plasma, platelets), total ischemic time of the first and second implanted lung, and surgical time. The total ischemic time was defined as the interval between cardiac arrest for DCD, or cross-clamp for DBD, until lung reperfusion in the recipient, hence including both cold and warm ischemic times. Surgical time was defined as the time from the initial incision to final skin closure.

The postoperative outcomes were as follows: need for ECMO, time on the ventilator, extubation status (successful first extubation, reintubation, tracheostomy, death before extubation), PGD at 0/24/48/72 h after LTx, Clavien–Dindo score, ICU stay, and hospital stay. PGD was based on the International Society for Heart and Lung Transplantation (ISHLT) consensus definition and was assessed by pulmonary edema on chest x-ray and PaO2/FiO2 at 0/24/48/72 h post-LTx (19). PGD grade 3 (PGD3) was assigned when the chest x-ray revealed pulmonary edema with a PaO2/FiO2 <200 or when the combination of ECMO with bilateral pulmonary edema on chest x-ray occurred. Data on arterial blood gases were acquired by automated extraction of electronic patient files. The chest x-rays were evaluated retrospectively by two experienced physicians. Postoperative surgical complications were graded according to the Clavien–Dindo classification (20). The longer-term outcomes included follow-up and patient survival.

### 2.4 Lung transplant procedure and immunosuppression protocol

In Leuven, a routine LTx is performed via bilateral anterolateral thoracotomy in a sequential single LTx fashion, with selective use of ECMO to anticipate and overcome hemodynamic and respiratory instabilities (hypercapnia, PGD, pulmonary hypertension, ventilatory limitations). In Prague, the surgical approach involves a clamshell thoracotomy with sequential single LTx, and protocol use of intraoperative central venoarterial (VA) ECMO. In case of a single LTx, in both centers a unilateral anterolateral thoracotomy is performed without ECMO.

Immunosuppression consisted of triple therapy with tacrolimus, mycophenolate mofetil, and steroids. The induction immunosuppression used was rabbit antithymocyte globulin (Leuven) or basiliximab (Prague).

### 2.5 Statistical analysis

Descriptive statistical analyses were performed using Microsoft 365 Excel (Windows). The graphs were plotted with GraphPad Prism10 (San Diego, CA, USA). The continuous variables were summarized as median (range: minimum–maximum) values, and the categorical variables as observed frequencies and percentages.

## 3 Results

### 3.1 Recipient and donor characteristics

A total of 160 patients underwent LTx in Leuven (*n* = 85; 53.1%) or Prague (*n* = 75; 46.9%). CHS storage was carried out in 36 LTx cases: 24 (66.7%) in Leuven and 12 (33.3%) in Prague. Most of the patients were men (*n* = 21; 58.3%) aged 61 (15–68) years. The BMI was 26.2 (13.5–29.9) kg/m^2^. Indications for LTx were as follows: 17 (47.2%) COPD, 14 (38.9%) ILD, 3 (8.3%) PVD, 1 (2.8%) CF, and 1 (2.8%) CLAD-BOS after LTx. Time on the waiting list was 97.5 (1–826) days. A total of 10 (27.8%) patients were transplanted in a HU setting, following pretransplant hospitalization of 9.5 (2–106) days, with 3 (8.3%) preoperative venovenous (VV) ECMO.

The donor population was predominantly female (*n* = 20; 55.6%), aged 56 (29–94) years, with a BMI of 25.6 (18.0–34.9) kg/m^2^. There were 28 DBD (77.8%) and 8 (22.2%) DCD procedures, of which 7 were DCD class 3 (DCD-III) and 1 DCD-IV. The causes of death were varied, with 19 (52.8%) patients dying because of intracerebral hemorrhage, 6 (16.7%) trauma [head injury (*n* = 1) and falling (*n* = 5)], 5 (13.9%) cardiac arrests, 3 (8.3%) cerebral ischemia, 2 (5.6%) suicide [drug intoxication (*n* = 1) and gunshot (*n* = 1)], and 1 (2.8%) status epilepticus. Preoperative ICU stay was 3 (1–12) days. PaO2/FiO2 was 413.5 (264–545). Oto-score for secretions was 1 and chest x-ray findings 0, indicating minor secretions and absence of opacities, respectively. The recipient and donor characteristics are presented in Table 1 and the Supplementary Material.

**Table 1:** Recipient and donor characteristics.

	Recipient					Donor				
	Sex (M/F)	Age (years)	Indication for LTx	Time on waiting list (days)	High urgency	Sex (M/F)	Age (years)	Type of donation	ICU stay (days)	PaO2/FiO2
1	M	55	ILD	121	Yes	M	44	DCD-III	12	457
2	F	15	CF	77	No	M	37	DBD	4	374
3	M	66	COPD	515	No	M	35	DCD-III	6	326
4	M	63	ILD	277	Yes	F	63	DBD	2	420
5	F	66	COPD	826	No	F	54	DCD-III	2	486
6	F	53	PVD^a^	500	Yes	F	68	DBD	1	448
7	M	66	ILD	56	No	M	44	DBD	3	361
8	F	64	COPD	651	No	F	59	DCD-III	5	376
9	M	54	ILD	297	No	M	54	DCD-IV	9	264
10	F	64	ILD	235	No	F	74	DBD	4	339
11	F	62	COPD	57	No	F	56	DBD	2	340
12	M	61	COPD	460	No	M	94	DCD-III	8	440
13	F	64	COPD	261	No	F	71	DBD	4	471
14	M	63	COPD	20	No	M	46	DBD	2	321
15	M	22	PVD^a^^b^	27	No	M	49	DBD	4	463
16	F	59	COPD	507	No	F	75	DBD	3	513
17	F	63	COPD	161	No	F	30	DBD	2	442
18	F	52	ILD	18	No	F	87	DBD	3	486
19	F	61	COPD	168	No	F	70	DBD	2	276
20	M	59	PVD^a^^c^	2	Yes	M	69	DBD	7	345
21	F	64	COPD	7	No	F	67	DBD	2	396
22	M	48	ILD	6	Yes	M	60	DBD	3	391
23	M	53	ILD	11	Yes	M	68	DBD	1	515
24	M	55	COPD	320	No	M	71	DCD-III	8	421
25	M	56	ILD	227	No	F	56	DBD	4	435
26	M	61	COPD	9	No	M	45	DBD	1	545
27	M	68	ILD	483	No	F	35	DBD	1	407
28	M	49	ILD	1	Yes	F	65	DBD	2	327
29	M	65	COPD	88	No	M	29	DBD	3	377
30	M	47	COPD	107	Yes	M	48	DCD-III	3	427
31	M	68	ILD	26	Yes	F	59	DBD	1	363
32	F	56	ILD	113	No	F	66	DBD	5	465
33	F	60	CLAD-BOS	48	Yes	F	44	DBD	2	390
34	F	65	COPD	72	No	F	36	DBD	7	431
35	M	57	ILD	3	No	F	54	DBD	4	510
36	M	67	COPD	60	No	M	40	DBD	12	337
MV	—	61	—	97.5	—	—	56	—	3	413.5

CF, cystic fibrosis; CLAD-BOS, chronic lung allograft dysfunction bronchiolitis obliterans syndrome; COPD, chronic obstructive pulmonary disease; DBD, donation after brain death; DCD, donation after circulatory death; F, female; FiO2, fraction of inspired oxygen; ICU, intensive care unit; ILD, interstitial lung disease; LTx, lung transplantation; M, male; MV, median value; PaO2, partial oxygen pressure; PVD, pulmonary vascular disease.

^a^ Chronic thromboembolic pulmonary hypertension.

^b^ Pulmonary capillary hemangiomatosis.

^c^ End-stage sarcoidosis with secondary pulmonary hypertension.

### 3.2 Controlled hypothermic storage

Indications for using the CHS device were overnight bridging (*n* = 26; 72.2%), remote procurement (*n* = 4; 11.1%), rescue allocation (*n* = 2; 5.6%), logistics (*n* = 2; 5.6%), feasibility (*n* = 1; 2.8%), and extended-criteria donor (*n* = 1; 2.8%) (advanced donor age: 94 years). Preservation temperature was 6.5°C (3.7°C–9.3°C), and CHS preservation time was 11 h 18 (02 h 42–17 h 09) and 13 h 40 (04 h 05–19 h 36) for the first and second implanted lungs, respectively. The distance between donor and recipient center was 148 (0–980) km, with air transport in 10 (27.8%) cases. The details on lung preservation are summarized in Table 2.

**Table 2:** Controlled hypothermic storage device and lung transplantation characteristics.

	Controlled hypothermic storage device			Lung transplantation details					
	Indication for CHS device	Preservation temperature (°C)	Preservation time CHS device first/second lung (h)	Type of LTx	Surgical approach	Intraoperative ECMO	Blood products (units)	Total ischemic time first/second lung (h)	Surgical time (h)
1	Feasibility	6.9	04 h 30/06 h 44	BLTx	BAT	No	0	07 h 31/09 h 17	07 h 27
2	Overnight bridging	5.9	07 h 56/11 h 20	Lobar BLTx	BAT	No	4	11 h 26/14 h 29	08 h 25
3	Overnight bridging	5.8	09 h 13/12 h 05	BLTx	BAT	Yes	6	11 h 35/14 h 40	07 h 20
4	Remote procurement	4.0	07 h 46/10 h 06	BLTx	BAT	Yes	20	10 h 42/13 h 12	11 h 01
5	Overnight bridging	6.1	12 h 42/15 h 38	BLTx	BAT	No	3	15 h 53/18 h 55	06 h 36
6	Overnight bridging	6.3	14 h 15/18 h 23	BLTx	BAT	Yes	1	17 h 43/22 h 02	12 h 12
7	Rescue allocation	6.4	06 h 03/NA	Single LTx	UAT	No	0	09 h 11/NA	03 h 23
8	Overnight bridging	8.8	12 h 15/15 h 20	BLTx	BAT	No	2	13 h 58/17 h 18	13 h 19
9	Overnight bridging	9.3	12 h 12/14 h 20	BLTx	BAT	No	0	14 h 59/16 h 51	05 h 39
10	Overnight bridging	5.7	07 h 43/09 h 07	BLTx	BAT	No	0	09 h 41/10 h 59	04 h 43
11	Remote procurement	8.9	04 h 36/06 h 32	BLTx	BAT	No	0	06 h 59/08 h 39	04 h 31
12	Extended-criteria donor^a^	6.9	02 h 42/04 h 20	BLTx	BAT	No	1	05 h 05/06 h 52	05 h 07
13	Overnight bridging	7.0	08 h 40/11 h 58	BLTx	BAT	Yes	3	12 h 03/15 h 23	08 h 35
14	Overnight bridging	7.6	12 h 40/15 h 44	BLTx	BAT	No	0	15 h 41/18 h 53	07 h 10
15	Overnight bridging	6.4	13 h 52/16 h 59	BLTx	BAT	Yes	5	16 h 40/19 h 45	07 h 39
16	Overnight bridging	5.0	14 h 11/16 h 08	BLTx	BAT	No	0	16 h 49/20 h 03	07 h 04
17	Logistics	9.0	04 h 14/05 h 38	BLTx	BAT	No	0	06 h 32/08 h 50	05 h 00
18	Overnight bridging	4.2	15 h 59/17 h 36	BLTx	BAT	No	0	18 h 05/19 h 48	06 h 09
19	Overnight bridging	5.4	13 h 14/16 h 57	BLTx	BAT	No	1	16 h 11/20 h 15	09 h 48
20	Overnight bridging	8.7	11 h 42/16 h 42	BLTx	BAT	No	0	15 h 27/20 h 24	12 h 29
21	Overnight bridging	8.6	13 h 27/15 h 01	BLTx	BAT	Yes	0	15 h 40/17 h 55	05 h 27
22	Overnight bridging	6.6	17 h 09/19 h 36	BLTx	BAT	Yes	14	19 h 44/22 h 48	08 h 21
23	Overnight bridging	6.5	11 h 43/15 h 47	BLTx	BAT	No	6	15 h 05/18 h 59	09 h 01
24	Overnight bridging	5.5	15 h 40/18 h 02	BLTx	BAT	No	0	18 h 03/21 h 16	07 h 23
25	Overnight bridging	8.1	10 h 30/12 h 32	BLTx	Clamshell	Yes	0	12 h 35/14 h 32	06 h 30
26	Overnight bridging	9.0	13 h 35/15 h 50	BLTx	Clamshell	Yes	4	16 h 25/18 h 28	08 h 11
27	Rescue allocation	5.5	10 h 50/NA	Single LTx	UAT	No	0	13 h 32/NA	02 h 57
28	Overnight bridging	6.9	11 h 45/13 h 35	BLTx	Clamshell	Yes	0	14 h 00/15 h 48	07 h 00
29	Remote procurement	3.7	03 h 07/04 h 05	BLTx	Clamshell	Yes	2	04 h 51/05 h 54	05 h 05
30	Overnight bridging	6.4	12 h 19/13 h 55	BLTx	Clamshell	Yes	2	14 h 37/15 h 52	04 h 02
31	Overnight bridging	9.0	10 h 50/12 h 38	BLTx	Clamshell	Yes	0	13 h 19/15 h 04	06 h 40
32	Overnight bridging	5.6	09 h 49/11 h 26	BLTx	Clamshell	No	0	11 h 50/13 h 45	05 h 55
33	Remote procurement	5.9	11 h 45/13 h 45	BLTx	Clamshell	Yes	20	13 h 44/15 h 35	08 h 00
34	Logistics	7.6	10 h 34/12 h 27	BLTx	Clamshell	Yes	0	13 h 14/14 h 16	05 h 20
35	Overnight bridging	6.7	08 h 32/11 h 50	BLTx	BAT	Yes	2	11 h 38/14 h 00	07 h 00
36	Overnight bridging	6.0	10 h 55/12 h 27	BLTx	BAT	Yes	2	12 h 53/14 h 37	05 h 45
MV	—	6.5	11 h 18/13 h 40	—	—	—	0.5	13 h 38/15 h 41	07 h 00

BAT, bilateral anterolateral thoracotomy; BLTx, bilateral lung transplantation; CHS, controlled hypothermic storage; ECMO, extracorporeal membrane oxygenation; LTx, lung transplantation; MV, median value; NA, not applicable; UAT, unilateral anterolateral thoracotomy.

^a^ 94-year-old male donor.

### 3.3 Surgical variables

Most recipients underwent a full-size bilateral LTx (*n* = 33; 91.7%), while two (5.6%) a single LTx, and one (2.8%) a lobar bilateral LTx (pediatric CF). Bilateral anterolateral thoracotomy was the surgical approach in 25 (69.4%) patients, clamshell in 9 (25%), and a unilateral anterolateral thoracotomy in 2 (5.6%). In 18 (50%) patients intraoperative ECMO was used, of which 1 (2.8%) was due to reperfusion edema of the first implanted lung. Altogether 19 (52.8%) patients required blood products intraoperatively [0.5 (0–20) units]. Total ischemic time was 13 h 38 (04 h 51–19 h 44) and 15 h 41 (05 h 54–22 h 48) for the first and second implanted lungs, respectively. The surgical time was 07 h 00 (02 h 57–13 h 19). The surgical variables are listed in Table 2 and the Supplementary Material.

### 3.4 Short-term outcomes

**Figure 1:**
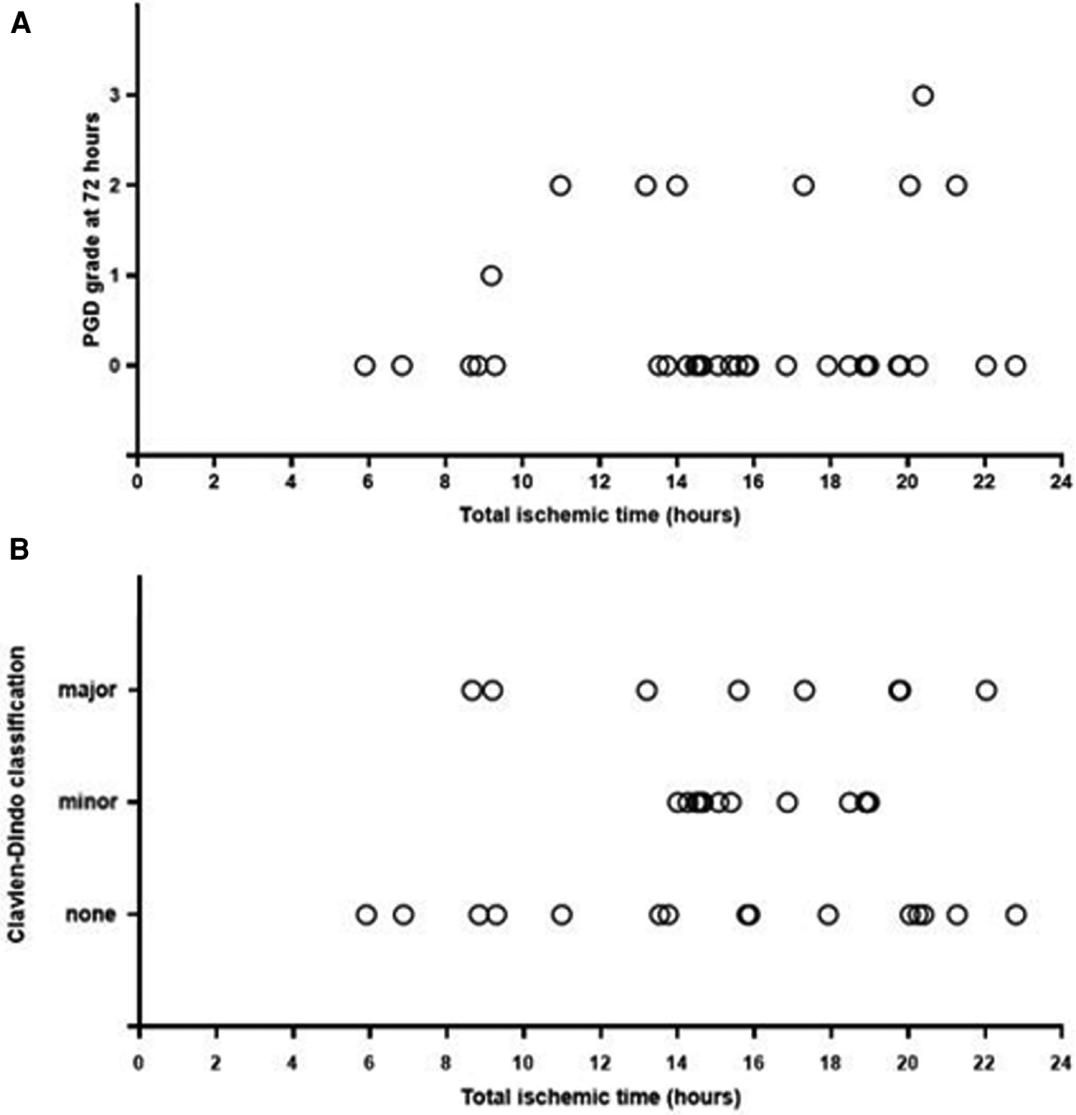
(**A**) Longest total ischemic time (h) and PGD grade (0–1–2–3) at 72 h. (**B**) Longest total ischemic time (hours) and Clavien–Dindo classification (none: grade 1–2, minor: grade 3a–3b, major: grade 4a–4b–5).

Five (13.9%) patients required ECMO postoperatively of which two (5.6%) for suboptimal oxygenation and ventilation caused by lung edema, and three (8.3%) for non-hypoxic reasons. One (2.8%) patient was switched from VV ECMO to VA ECMO due to cardiogenic shock. She died at POD7 after withdrawal of supportive therapy because of irreversible ischemic-hypoxic encephalopathy. Postoperative time on ventilator was 25.5 (6–526) h. Two (5.6%) patients required tracheostomy due to failure from weaning. Within and at 72 h PGD3 was present in 12 (33.3%) patients and one (2.8%) patient, respectively (Figure 1A). ICU stay was 8 (4–62) days, while hospital stay was 28 (13–87) days. During hospitalization, six (17%) patients suffered from a Clavien–Dindo 3b scoring, and seven (19%) from a 4a classification (Figure 1B). Follow-up was 139 (7–446) days. At the final date of follow-up, three (8.3%) patients were still hospitalized, and patient survival was 97.2% (*n* = 35). The postoperative outcomes are outlined in Table 3 and the Supplementary Material.

**Table 3:** Postoperative outcomes.

	Postoperative ECMO	Time on ventilator (h)	PGD at 0/24/48/72 h	ICU stay (days)	Hospital stay (days)	Clavien–Dindo score
1	No	37	0/0/0/0	5	19	2
2	No	44	3/2/0/0	14	26	3a
3	No	42	0/0/0/0	6	31	3a
4	No	NA	3/3/3/2	62	87	4a
5	No	48^a^	0/0/0/0	9	30	3b
6	Yes	108	UG/UG/UG/0	16	32	4a
7	No	16	0/0/2/1	4	18	4a
8	Yes	NA	3/3/3/2	7^b^	7^b^	5
9	No	29	3/2/0/0	8	16	3b
10	No	42	0/2/2/2	7	21	2
11	No	19	1/0/0/0	7	83	4a
12	No	15	0/0/0/0	4	31	2
13	Yes	86	3/3/3/0	15	45	3a
14	No	22	0/0/1/0	5	30	3a
15	Yes	64	UG/UG/UG/0	14	34	4a
16	No	20	3/2/2/2	8	28	2
17	No	19	0/0/0/0	7	40	2
18	No	49	0/0/3/0	12	27	4a
19	No	57	3/0/1/0	9	24	2
20	No	17	2/2/3/3	5	20	2
21	No	18	0/0/0/0	7	19	2
22	Yes	39	2/3/2/0	8	Ongoing	2
23	No	57	1/3/0/0	Ongoing	Ongoing	3b
24	No	19	2/1/3/2	Ongoing	Ongoing	2
25	No	6	0/0/0/0	11	20	3a
26	No	7	0/0/0/0	11	34	3b
27	No	10	0/0/0/0	7	13	2
28	No	17	0/0/0/0	7	20	2
29	No	31	0/0/0/0	11	19	2
30	No	21	0/0/0/0	8	19	2
31	No	41	0/0/0/0	46	52	3b
32	No	8	0/0/0/0	7	20	2
33	No	526^a^	0/0/2/0	44	51	4a
34	No	12	0/0/0/0	9	34	3a
35	No	47	2/1/1/2	14	26	3a
36	No	6	0/0/0/0	20	32	3b
MV	—	25.5	—	8	28	—

ECMO, extracorporeal membrane oxygenation; ICU, intensive care unit; PGD, primary graft dysfunction; MV, median value; UG, ungradable.

^a^ Tracheostomy due to failure from weaning.

^b^ Death on day 7 postoperatively.

## 4 Discussion

This first European experience with the portable LUNGguard shows safe and good short-term outcome for preservation through CHS, and the possibility of converting the transplant procedure to a diurnal activity. The median preservation temperature was 6.5°C. The main indication for using CHS was overnight bridging (*n* = 26; 72.2%). Although maximum preservation (19 h 36) and total ischemic times (22 h 48) importantly exceeded the current limits of 8 and 10 h, respectively, PGD3 incidence at 72 h was 2.8%.

The hypothesis suggesting CHS is associated with improved post-reperfusion outcome compared with SIS was first proposed by the group of Joel Cooper (Toronto) between 1989 and 1993, based on animal research on donor lung preservation temperature (11–14, 21–23). The conclusion of this preclinical research was that the optimal lung preservation temperature was around 8–10°C, allowing the option of extended preservation up to 24 h (11, 21). Three decades later, in 2021, there was a reinstated interest in preservation temperature by Ali et al. and Cypel et al. from the Toronto group, with a first clinical evaluation of five patients receiving LTx after a CHS at 10°C (4). The purpose was overnight bridging and starting the LTx procedure in the morning. Preservation time was 10 h 24 (09 h 55–14 h 48) and 12 h 06 (10 h 54–16 h 30) for the first and second implanted lung, respectively. There was no PGD3 at 72 h, median time on the ventilator was 2 (0–7) days, median hospital stay was 17 (14–26) days, and 30-day survival was 100%. Based on these promising results, the research group led by Cypel set up a prospective non-randomized clinical trial assessing the extension of the static donor lung preservation at 10°C (*n* = 70) vs. SIS (*n* = 140) (9). The lungs were procured and transported in an ice cooler for 03 h 30 [interquartile range (IQR), 02 h 18–04 h 09], and after arrival in the recipient center, they were preserved in a 10°C temperature-controlled incubator for 07 h 48 (IQR, 05 h 46–09 h 37) until implantation. Preservation time (including lung implantation in this study) was 12 h 28 (IQR, 10 h 14–14 h 12) and 14 h 09 (IQR, 12 h 03–15 h 45) for the first and second implanted lungs, respectively. PGD3 incidence at 72 h was 5.7% vs. 9.3%, and 1-year patient survival was 94% vs. 87%, for the CHS vs. SIS groups, respectively. Minor differences were observed in ICU stay (5 vs. 5 days), hospital stay (25 vs. 30 days), and need for postoperative ECMO (5.7% vs. 9.3%).

In February 2022, the commercially portable CHS device LUNGguard was first implemented in North America. Specifically for this device, the clinical non-randomized post-market registry study “Global Utilization And Registry Database for Improved preservAtion of doNor LUNGs’ (GUARDIAN-LUNG) (NCT04930289) was started, with the objective of comparing the outcomes after LTx by CHS vs. SIS (24, 25). Preliminary registry data about the North American experience was presented at ISHLT 2023, enrolling 86 LUNGguard and 90 SIS patients. The median preservation temperature was 4.9°C, total ischemic time was 7 h 26 ± 1 h 51, and PGD3 incidence at 72 h was 8.1% (7/86). The CHS cohort had a clinically relevant 54% reduction in PGD3 incidence at 72 h (*p* = 0.058) compared with SIS, and was also associated with significantly improved 1-year estimated patient survival [CHS 92.7% vs. SIS 82.2% (*p* = 0.02)] (26).

Our manuscript describes the implementation of this CHS device over a 14-month period in two European centers. It is worth mentioning that the preservation temperature in our cohort was higher compared with the GUARDIAN-LUNG (6.5°C vs. 4.9°C). Both Leuven and Prague attempt to reach higher temperatures, based on the favorable outcome of lungs preserved at 10°C (4, 8, 9). Therefore, we adopted a strategy in which we use maximum 250 g of ice on the bench table, and target a donor lung surface temperature between 8°C and 12°C prior to storage in the CHS device. We observed that the starting surface temperature of the donor lung directs the average preservation temperature afterward: starting temperatures >10°C vs. 8–10°C vs. <8°C were associated with average preservation temperatures above 8°C vs. 6–8°C vs. 4–6°C, respectively. The duration of preservation also influences the temperature curve, which changed during storage, finding equilibrium around 6°C.

Another major difference from the GUARDIAN-LUNG analysis concerns total ischemic time. Compared with the registry with total ischemic time of 7 h 26 ± 1 h 51, we report considerably longer total ischemic times with 15 h 41 (05 h 54–22 h 48) for the second implanted lung. In 52.8% (*n* = 19) of the patients, total ischemic times exceeded 15 h, with a maximum of 22 h 48. Nevertheless, PGD3 at 72 h was only 2.8% in our series. The promising findings of this first European experience with extended preservation and total ischemic times have encouraged Leuven and Prague to implement and standardize overnight bridging (*n* = 26; 72.2%) allowing a shift toward transplantation during the daytime. The literature suggests that nocturnal transplantation might be associated with worse outcomes because of limited resources, shortage of personnel, and lesser technical expertise (27–32). Moreover, fatigue and sleep deprivation of the LTx team might have a negative repercussion on the cognitive and psychomotor skills. Accordingly, the LTx policies of Leuven and Prague have considerably changed, with focus on flexibility and overnight bridging when donor cross-clamp time is planned after 6:00 PM. After procurement, the lungs are stored in the CHS device unattended in the surgical theater of the recipient center. Patient induction occurs the next day at 7:30 AM, and the LTx is performed during the daytime in optimal conditions with a well-rested team and maximal medical expertise. In addition, extended preservation and ischemic times allow expansion of the donor pool through long-distance lung procurement, rescue allocation, facilitation of immunological crossmatch test, and acceptance of a second pair of donor lungs in case of simultaneous or overlapping LTx.

Last but not least, we reported a higher number of DCD procedures (*n* = 8; 22.2%) compared with the registry [CHS *n* = 15/85 (17.6%) and SIS *n* = 6/90 (6.7%)] (26). These findings can be attributed to the rapidly growing experiences of Leuven with DCD procedures (Leuven *n* = 7 vs. Prague *n* = 1). In fact, based on the favorable long-term survival in DCD-III and DBD lung donor recipients, as reported by ISHLT in 2019, Leuven increasingly performs DCD procurements to expand the donor pool (33).

Several questions on CHS remain, concerning the ideal temperature, extended vs. short preservation, long-term outcome, and potential benefits for extended-criteria donors. Randomized controlled trials (RCTs) and a propensity-matched study from a large GUARDIAN-LUNG cohort (*n* = 500) are awaited (24, 25). Furthermore, a multicenter RCT is currently being conducted by Toronto (X°Port Lung Transport Device, Traferox Technologies Inc.): “Safety of 10°C Lung Preservation Versus Standard of Care: A Multi-Center Prospective Non-Inferiority Trial” (NCT05898776), comparing 160 CHS vs. 160 SIS (24, 34) cases.

## 5 Conclusion

CHS by LUNGguard seems feasible and safe, despite the relatively high-risk recipient and donor profiles (DCD 22.2%) and extended preservation periods. PGD3 at 72 h of 2.8% was observed in this series. The CHS technology potentially allows overnight bridging and shifting toward daytime transplantation, optimizing working conditions. However, several questions remain and multicenter randomized trials are awaited.

## Data Availability

The original contributions presented in the study are included in the article/**Supplementary Material**; further inquiries can be directed to the corresponding author.
