# Supplementary material for: Lung transplantation following controlled hypothermic storage with a portable lung preservation device: first multicenter European experience
Source: Front Cardiovasc Med. 2024 Jun 6;11:1370543. doi: 10.3389/fcvm.2024.1370543 (PMC11187339; doi:10.3389/fcvm.2024.1370543)
Supplement: Supplementary file 1 [file Table1.docx]

**Supplementary material 1. Extracorporeal membrane oxygenation in the preoperative, intraoperative and postoperative setting**

| µ | Preoperative | | Intraoperative | | Postoperative | |
| --- | --- | --- | --- | --- | --- | --- |
|  | **ECMO** | **Indication** | **ECMO** | **Indication** | **ECMO** | **Indication** |
| 1 | No | / | No | / | No | / |
| 2 | No | / | No | / | No | / |
| 3 | No | / | VA  (peripheral) | Cardiac arrest | No | / |
| 4 | VV  (peripheral) | Respiratory failure | VA  (peripheral) | Continuation pretransplant bridging and PHT | No | / |
| 5 | No | / | No | / | No | / |
| 6 | No | / | VA  (central) | Small chest cavity with insufficient exposure | VV  (peripheral) | Suboptimal ventilation and oxygenation with underlying bidirectional shunting interatrial septum stent. Medical history in 2020 of placement interatrial septum stent for right ventricle failure in the context of severe chronic thromboembolic pulmonary hypertension and atrial septal defect |
| 7 | No | / | No | / | No | / |
| 8 | No | / | No | / | VA and VV ECMO (peripheral) | VV ECMO for reperfusion oedema. Temporary VA ECMO due to hemodynamic collapse caused by cannula misplacement with iliac vein dissection and retroperitoneal hematoma |
| 9 | No | / | No | / | No | / |
| 10 | No | / | No | / | No | / |
| 11 | No | / | No | / | No | / |
| 12 | No | / | No | / | No | / |
| 13 | No | / | VV  (peripheral) | Reperfusion oedema firstly implanted graft | VV  (peripheral) | Reperfusion oedema |
| 14 | No | / | No | / | No | / |
| 15 | No | / | VA  (peripheral) | Cardiac arrest | VA  (peripheral) | Right and left ventricle failure |
| 16 | No | / | No | / | No | / |
| 17 | No | / | No | / | No | / |
| 18 | No | / | No | / | No | / |
| 19 | No | / | No | / | No | / |
| 20 | Np | / | No | / | No | / |
| 21 | No | / | VVA  (VV peripheral and A central) | Continuation pretransplant bridging and PHT | No | / |
| 22 | VV (peripheral) | Respiratory failure | VV (peripheral) | Continuation pretransplant bridging | VV  (peripheral) | Suboptimal ventilation and oxygenation |
| 23 | VV  (peripheral) | Respiratory failure | No | / | No | / |
| 24 | No | / | No | / | No | / |
| 25 | No | / | VA  (central) | Protocol | No | / |
| 26 | No | / | VA  (central) | Protocol | No | / |
| 27 | No | / | No | / | No | / |
| 28 | No | / | VA  (central) | Protocol | No | / |
| 29 | No | / | VA  (central) | Protocol | No | / |
| 30 | No | / | VA  (central) | Protocol | No | / |
| 31 | No | / | VA  (central) | Protocol | No | / |
| 32 | No | / | No | / | No | / |
| 33 | No | / | VA  (central) | Protocol | No | / |
| 34 | No | / | VA  (central) | Protocol | No | / |
| 35 | No | / | VA  (central) | Protocol | No | / |
| 36 | No | / | VA  (central) | Protocol | No | / |

A: arterial, ECMO: extracorporeal membrane oxygenation, LTx: lung transplantation, PHT: pulmonary hypertension, VA: venoarterial, VV: venovenous, VVA: venovenoarterial
