# Supplementary material for: Lung transplantation following controlled hypothermic storage with a portable lung preservation device: first multicenter European experience
Source: Front Cardiovasc Med. 2024 Jun 6;11:1370543. doi: 10.3389/fcvm.2024.1370543 (PMC11187339; doi:10.3389/fcvm.2024.1370543)
Supplement: Supplementary file 2 [file Table2.docx]

## **Supplementary material 2. Postoperative complications according to the Clavien-Dindo score**

|  | 3a | 3b | 4a | 4b | 5 |
| --- | --- | --- | --- | --- | --- |
| 1 | NA | NA | NA | NA | NA |
| 2 | Exsufflation of pneumothorax | NA | NA | NA | NA |
| 3 | Pleural puncture for pleural effusion | NA | NA | NA | NA |
| 4 | Minitracheostomy | Open tracheostomy for failure from weaining | Acute renal failure requiring CVVH and intermittent hemodialysis. CO2-retention after bronchoscopy requiring intubation and ICU-monitoring. | NA | NA |
| 5 | Pleural puncture for pleural effusion | Electrical reconversion of atrial fibrillation | NA | NA | NA |
| 6 | NA | NA | Acute renal failure requiring CVVH | NA | NA |
| 7 | NA | NA | Acute mixed aphasia requiring ICU-monitoring. | NA | NA |
| 8 | NA | NA | NA | NA | Ischemic-hypoxic encephalopathy with infaust neurological prognosis, resulting in withdrawal of supportive therapy at POD7 |
| 9 | NA | Exploratory thoracotomy for unilateral hemothorax at POD1. | NA | NA | NA |
| 10 | NA | NA | NA | NA | NA |
| 11 | NA | Carotid thrombectomy for an ischemic stroke. Aortic root thrombectomy for high embolization risk thrombus in the aortic root. | Stroke unit monitoring after ischemic stroke | NA | NA |
| 12 | NA | NA | NA | NA | NA |
| 13 | Pleural puncture of a pleural collection. | NA | NA | NA | NA |
| 14 | Repositioning chest tube because of dislocation with resulting pneumothorax. | NA | NA | NA | NA |
| 15 | NA | Wound debridement thoracotomy.  Pericardial effusion drainage. | ICU-monitoring for pericardial effusion. | NA | NA |
| 16 | NA | NA | NA | NA | NA |
| 17 | NA | NA | NA | NA | NA |
| 18 | Minitracheostomy | NA | Reintubation for failure from weaning. | NA | NA |
| 19 | NA | NA | NA | NA | NA |
| 20 | NA | NA | NA | NA | NA |
| 21 | NA | NA | NA | NA | NA |
| 22 | NA | NA | NA | NA | NA |
| 23 | NA | Exploratory thoracotomy for unilateral hemothorax at POD1. | NA | NA | NA |
| 24 | NA | NA | NA | NA | NA |
| 25 | Bilateral pleural puncture for pleural effusion. | NA | NA | NA | NA |
| 26 | NA | Chest tube for pneumothorax. | NA | NA | NA |
| 27 | NA | NA | NA | NA | NA |
| 28 | NA | NA | NA | NA | NA |
| 29 | NA | NA | NA | NA | NA |
| 30 | NA | NA | NA | NA | NA |
| 31 | Bronchoscopy for left lower lobe atelectasis due to mucus plug. | Electrical cardioversion for atrial fibrillation. | NA | NA | NA |
| 32 | NA | NA | NA | NA | NA |
| 33 | Chest tube for pleural effusion. | Open tracheostomy for failure from weaning. | Graft dysfunction.  Pneumonia. | NA | NA |
| 34 | Chest tube for pleural effusion. | NA | NA | NA | NA |
| 35 | Pleural puncture for pleural effusion.  Chest tube for pneumothorax. | NA | NA | NA | NA |
| 36 | NA | Electrical cardioversion for atrial fibrillation | NA | NA | NA |

CO2: carbon dioxide, CVVH: continuous venovenous hemofiltration, ICU: intensive care unit, NA: not applicable, POD: postoperative day
